# Community ownership of biopsychosocial model of care: a qualitative study in the Katana health district, Democratic Republic of Congo

**DOI:** 10.1080/16549716.2025.2555030

**Published:** 2025-09-04

**Authors:** Bertin Mutabesha Kasongo, Christian Eboma Ndjangulu Molima, Hermès Karemere, Samuel Lwamushi Makali, Landry Chahihabwa Mugisho, Albert Tambwe Mwembo, Ghislain Balaluka Bisimwa, Abdon Mukalay Wa Mukalay

**Affiliations:** a Ecole Régionale de Santé Publique (ERSP), Catholic University of Bukavu, Bukavu, Democratic Republic of Congo; b School of Public Health, University of Lubumbashi, Lubumbashi, Democratic Republic of Congo; c Faculty of Pharmaceutical Sciences and Public Health, Official University of Bukavu, Bukavu, Democratic Republic of Congo; d Centre de recherche sur les politiques et systèmes de santé (CR3-POLISSI), Ecole de santé publique, Université libre de Bruxelles, Brussels, Belgium; e Programme National des Maladies Non Transmissibles, Division Provinciale de la Santé du Sud-Kivu, Bukavu, Democratic Republic of Congo; f Département de Nutrition, Centre de Recherche en Sciences Naturelles, Lwiro, Democratic Republic of Congo; g Unité d’Epidémiologie Clinique et Pathologies Tropicales, Faculté de médecine, Université de Lubumbashi, Lubumbashi, Democratic Republic of Congo

**Keywords:** Community participation, ownership, primary health care, person centered care, biopsychosocial model, chronic diseases

## Abstract

**Background:**

The WHO recommends the Person-Centred Care approach, based on the biopsychosocial (BPS) model with community participation, to streamline the management of chronic diseases in Primary Health Care (PHC) activities and reduce their growing burden, even in Africa. The Democratic Republic of Congo’s (DRC) experience of community participation in implementing the BPS model for chronic diseases has been little explored.

**Objective:**

To describe community ownership of the biopsychosocial model of chronic disease care in PHC facilities in South Kivu province, DRC.

**Methods:**

A basic interpretive qualitative study was conducted in April 2024, in three health areas of the Katana health district, among beneficiaries of chronic disease interventions. Nine in-depth interviews were conducted with care providers and community representatives, followed by two focus groups with community health workers (CHWs) and a document review. An inductive-deductive content analysis was carried out using ATLAS.ti 24 software.

**Results:**

Based on the four main themes identified in the study, community ownership of the model depends on various factors (relational, organizational, and motivational/supportive). As perceptions, respondents recognized the model’s application through partner support, meetings with community representatives and patient decision-making. Community involvement in the model was observed through participatory meetings, reinforced home visits, psycho-education, and club solidarity. Significant challenges included the lack of training for providers and CHWs, CHW’s financial demotivation, poor dissemination of model, and patient poverty. Proposed strategies included a participatory stakeholder audit, capacity-building on the model, psychologist availability, and income-generating activities to motivate CHWs.

**Conclusions:**

Community ownership of the BPS model is a vital pillar to support effective and resilient chronic disease management, rationalizing it in PHC for better health outcomes. Healthcare systems should consider these identified factors in the policy definition and rationalization process for these diseases by establishing effective coordination mechanisms.

## Paper Context

- **Main findings:** Community ownership of biopsychosocial model interventions for chronic disease management depends on multiple factors including relational (solidarity in patient clubs), organizational (inclusive and participatory meetings, reinforced home visits, and capacity building on the biopsychosocial model), and motivational/supportive (psychoeducation and income-generating activities).
- **Added knowledge:** Given that the Democratic Republic of Congo’s experience with community participation in implementing the biopsychosocial model of chronic disease care has been little explored, this study provides further information for managing these diseases, promoting their prevention and control.
- **Global health impact for policy and action:** Taking into account the factors identified for this community ownership of the biopsychosocial model in the definition of health policies for chronic disease management will enable health systems to rationalize them in primary health care, by putting in place effective coordination mechanisms.

## Background

Healthcare systems are increasingly moving towards a Person-Centered Care (PCC) approach, where patients’ needs, preferences and values are at the center of decision-making [1]. Community participation, as a dynamic process in which populations become empowered to take charge of their health and improve it, appears to be a key element of this strategy. It involves them in the planning, implementation, and evaluation of health services [2].

The 2018 Astana Conference on Primary Health Care (PHC) focuses on community participation to achieve the Sustainable Development Goals (SDGs), particularly the health-related ones, in reducing morbidity and mortality from diseases and other health conditions, by promoting PHCs [3]. This type of PCC incorporates the biopsychosocial (BPS) model of care, which involves treating the patient holistically by offering biological, psychological, and social care tailored to the patient’s needs and values [4].

However, current data indicate that NCDs are becoming a growing concern worldwide, constituting the bulk of the disease burden, more specifically in Africa, alongside communicable diseases [5]. For this reason, the World Health Organization (WHO) recommends that greater attention be paid to them, and that they be rationalised in the implementation of PHC activities, to reduce their burden and ensure their prevention and control. In this process of rationalization, actions are based on the BPS model of care, while developing effective community involvement to ensure ownership [6]. With this BPS model, healthcare systems are gradually called upon to transform themselves from biomedical systems into biopsychosocial systems, more oriented towards PCC, in which people take part in making decisions about their health [7].

Some studies worldwide have explored community participation in implementing the PCC approach, particularly in the management of chronic diseases and the adoption of various strategies to enhance primary care practice and the population’s quality of life [8–11]. Multiple authors have sought to define community ownership, and provide complementary elements [12,13]. Community ownership is determined by four main dimensions of capacity, including leadership, governance and participation in decision-making; sustaining capacity through resource mobilization for empowerment and networking; managing the targeted intervention program; and engaging with state bodies and broader society to enforce rights and address stigma issues [12].

The organization of community participation in the Democratic Republic of the Congo (DRC), as mandated by the health system, is evident in the various health areas (HAs) of the health district (HD). Community health workers (CHWs) are grouped in community animation cells (CACs) at the village level. In health area development committees (HADCs) of health centers (HC), CHWs operate in collaboration with local leaders and healthcare providers [14].

The DRC’s experience with community participation reveals both successes and limitations in implementing health interventions, particularly in areas such as maternal and child health, communicable diseases, water, sanitation and hygiene (WASH) [15–17]. On the other hand, there is very little experience with community participation in the implementation of the PCC approach, minimally in the BPS model of care for non-communicable diseases (NCDs) [18].

In South Kivu province, the Katana health district is one of the three HDs benefiting from the NGO Louvain Cooperation’s support as part of the National Program for Non-Communicable Diseases (NPNCD), particularly in the management of diabetes mellitus, hypertension, and mental health, as an entry point for the PCC strategy, by implementing the BPS model.

Thus, this work aims to describe the community ownership of the BPS model of care in the first-level facilities of Katana HD, in South Kivu, DRC, through the management of chronic diseases.

## Methods

### Study settings

The present study is taking place in the province of South Kivu, specifically in three HAs in Katana HD, concerned by the support of ‘Louvain Cooperation’, within the context of the NPNCD, notably the management of chronic diseases (diabetes mellitus, hypertension, and mental health). These are the Birava, Kabushwa, and Mushweshwe HAs. This support from ‘Louvain Cooperation’ covers 2022 to 2026. As part of the implementation of the BPS model, this support consists of building the capacity of healthcare providers in the holistic management of chronic pathologies:

– The establishment of comprehensive patient files that integrate somatic, psychological, and social aspects, as well as the organization of case discussions between providers and referring physicians.
– With particular focus on the development of community strategies for effective and sustainable involvement of the community in the implementation of the model (capacity-building of CHWs and their involvement in sensitization campaigns, particularly on the prevention and control of diabetes and hypertension, strengthening collaboration between providers and the community, support for the activities of diabetic clubs, reinforcement of home visits, patient referrals, psychosocial support, etc.),
– The supply of essential medicines and equipment for effective treatment, supervision of the HD Executive Team, and coordination of the NPNCD in the South Kivu provincial health division.

**Figure 1.**
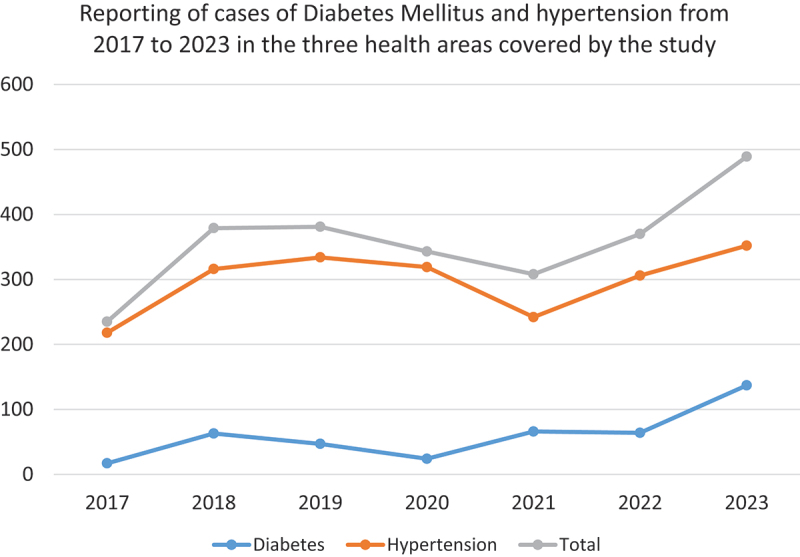
Trend in notified cases of chronic diseases (diabetes and hypertension) in the three HAs concerned by the study (source: DHIS2, 2017–2023, DRC).

Table 1 and Figure 1, respectively, show the descriptions of these three HAs and the trend in notifications of cases of chronic diseases (diabetes and hypertension). Note that the Katana HD benefited from a previous research and development project (PRD) from 2017 to 2019, which was financed by the Belgian cooperation (ARES-CCD). This project aimed to reorganize care at the HC level according to a person-centered approach, with chronic diseases as the entry point. This explains the increase in case notification over this period, as shown in Figure 1. Case reporting has increased again with the launch of the new ‘Louvain Cooperation’ project in the NPNCD in 2022.

**Table 1.** Description of the three HAs concerned in Katana health district in 2023.

Health Area	Population	Number of CACs	Curative Utilization Rate*
BIRAVA	30305	25	62%
KABUSHWA	14457	5	68%
MUSHWESHWE	14880	20	86%

Legend: CAC= Community Animation Cell, *(New Cases/Total Population) x 100 (Source: DHIS2, year 2023, DRC).

## Conceptual framework

**Figure 2.**
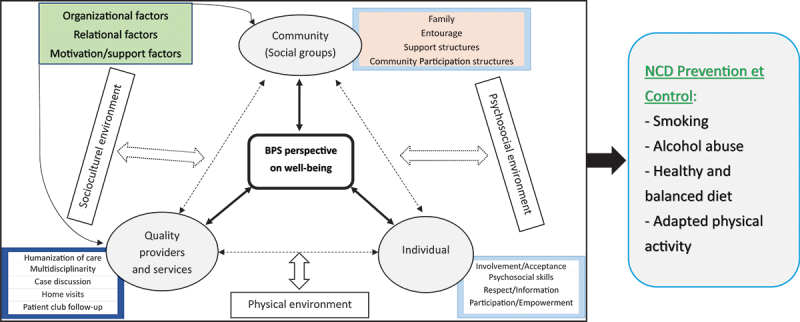
Conceptual framework of community participation in the implementation of the BPS model of care, for the management of NCDs (Bps = biopsychosocial), inspired by Harrison SR & Jordan AM [19].

We developed our conceptual model (Figure 2) to explain community involvement in the implementation of the BPS model of care, applicable to NCDs, drawing inspiration from Harrison SR & Jordan AM [19].

From an integrated biopsychosocial care perspective, providing person-centered care for chronic diseases requires interaction between quality healthcare providers and services, the person receiving comprehensive care (BPS), and the community (social groups) in which this person lives. Community interventions are based on the prevention, control, and promotion of NCDs. They mainly address risk factors such as tobacco, alcohol abuse, a healthy and balanced diet, regular physical activity, follow-up care under the guidance of healthcare providers, and community reference. These interventions, which define this interaction, are grouped into three main factors: organizational, relational and motivational/supportive factors.

– Quality healthcare providers and services: This dimension considers the physical environment (resources), which includes the availability of competent healthcare personnel for the provision of BPS care (holistic care oriented according to patients’ needs and preferences, etc.) and quality services (materials, equipment, and medicines). The humanization of care characterizes this component, including multidisciplinary, case discussions among providers, the organization of home visits, and support structures (patient clubs)
– Individual: the individual is responsible for his or her health (empowerment), in a psychosocial environment (acceptance, participation in his or her care, health education for behavior change, etc.)
– Community (social groups): The various social groups that interact with the person must support them in a specific socio-cultural context. These groups include the patient’s entourage, social networks (such as patient clubs), and other community participation structures (CHWs grouped into CACs, HADCs) that provide support to the patient and must interact with both the patient and the providers, during follow-up treatment. This component is crucial in implementing preventive and promotional activities to reduce and control NCDs.

This model is particularly relevant in the context of Katana care, as it integrates not only the individual as an active participant in their health, but also the critical role played by the community in providing care.

### Study design

This is a basic interpretive qualitative study [20,21]. It aims to describe community participation in managing chronic diseases and the ownership of the BPS model of care. The choice of this approach is justified not only by the fact that the subject is less well-known and developed in the DRC, but also because it allows us to explore the perceptions and experiences of healthcare providers and community representatives within the community dynamic surrounding this model, in the management of chronic diseases.

### Study participant selection

Our study focused on healthcare providers, who implement biopsychosocial care, as well as representatives of the HA community involved in the study in Katana HD. Participants were purposively selected, based on their experience with community participation, specifically as first-level caregivers and community representatives.

For the community, the selection criteria were: to be a member of the CAC and/or HADC of an HA concerned by the study in Katana HD; to be active in the implementation of community activities; to have at least two years of experience as CHWs; to have at least one person with chronic diseases in their family; to be present on the day of the interview and to agree to answer our questions.

For the healthcare providers, the selection criteria were: to be a caregiver in the health facility for the HA concerned by the study in the Katana HD; to have at least five years of experience as a provider in the HD and at least one year since the start of the ‘Louvain Coopération’ project; to be present on the day of the interview, and to agree to answer our questions.

### Data collection

Semi-structured in-depth interviews and Focus Groups (FGs) were conducted with study participants. A document review was also completed during this research. The reason for this combination of methods was to obtain in-depth and diversified information relevant to the study’s purpose (individual points of view, then information gathered through interaction between group members and document analysis) [22,23]. The data was collected in April 2024.

Data were collected using two interview guides explicitly designed for care providers and community representatives (Appendix 1 and Appendix 2), supplemented by a document review.

The first interview guide enabled us to collect data from providers in three HCs (one provider per HC). The interviews focused mainly on the organization of community participation in HA, their perceptions of this organization and its place in the implementation of the BPS model, the strengths and weaknesses of community participation in improving the provision of BPS care, the involvement of the community in decision-making regarding care that affects their health, the existence of discussion forums between community and caregivers concerning comprehensive care, according to the BPS model, the strategies to be proposed for the appropriation of the BPS model.

The second guide enabled us to collect data from 6 HADC board members (2 per HA). The interviews focused on their opinions of the relationship between healthcare providers and the community on the one hand, and between the community and patients on the other, describing this dynamic around the BPS care strategy, the role of the community (community participation structures, community leaders, etc.) in decision-making and the implementation of interventions in HA. This same guide was used to conduct two FGs with 16 CHWs, with 8 CHWs per FG (8 in the HA of Birava and 8 in the HA of Kabushwa), an average number of participants ideally recommended [24]. These FGs were much more focused on identifying challenges and proposing strategies for improving community participation related to the management and ownership of interventions aiming at the BPS model. Given that the HAs of Birava and Mushweshwe are nearby, and can share the same reality, unlike the HA of Kabushwa, which is very distant from the others, this justified the creation of these two FGs.

This selected sample was sufficient to provide important information about the process of community ownership of the BPS model in the structures concerned by the study, in Katana HD. It saturated the responses obtained.

In addition, a document review was also carried out to triangulate the information collected through individual interviews and FGs, by exploiting the NPNCD provincial coordination’s supervision reports on the implementation of activities in the Katana HD. Two supervision reports (Doc 1 and Doc 2) found at the HD central office were analyzed (for the 3^rd^ quarter of 2022 and the first quarter of 2023), focusing on community activities related to BPS care.

All data collection was conducted by the principal investigator, who is experienced in conducting qualitative inquiries. The researcher had no relationship with the study participants. During the interviews, the researcher remained neutral, allowing participants to express themselves freely without influencing their answers. These interviews began after obtaining the interviewees’ consent, following an explanation of the study’s purpose.

These individual interviews and FGs took place at the Health Center, in French or Swahili, in a room guaranteeing confidentiality, and lasted on average between 30 and 45 minutes for individual interviews and 1:00 to 1:30 for FGs. They were recorded using a SONY Dictaphone.

### Data analysis

Each interview and FG were then transcribed verbatim into a Word file, separately, by two independent persons. The Swahili interviews were translated into French, and vice versa, by two independent translators. The principal investigator, fluent in Swahili and French, then reviewed the translations and transcriptions by listening to the audio recordings again and rereading the transcripts simultaneously.

We conducted an inductive and deductive analysis of the interview content, utilizing ATLAS.ti 24 software to organize, structure and code the transcripts. This analysis was then continued manually to ensure the trustworthiness of the data [25]. The various individual interviews and FGs were first read through several times to familiarize ourselves with them. The codes were then gradually drawn out, intuitively, from what the respondents had to say, line by line, while relying on the interview guides. A consensus was reached to keep the list of codes together, following the triangulation of information from different sources, to enhance the trustworthiness of the results. Then, in a concerted effort, the researchers defined the sub-themes by progressively grouping similar codes. From these sub-themes, the main themes were retained. The results are presented according to the selected themes and their respective sub-themes, supported by citations. Elements from the document review (supervision reports) were considered in this analysis. The process was iterative and collaborative. First, the principal investigator (BMK) carried out the structural and line-by-line coding. He was then joined by two other researchers (CENM and SLM), who validated the codes, revisiting the transcripts as necessary, and defined the sub-themes and main themes under the supervision of a senior researcher (AMM), who had research experience and ensured the consistency of the analyses. Various team meetings were held with the senior researcher (supervisor) to harmonize viewpoints on the analysis approach and results. These meetings enabled us to set aside all presuppositions and remain as objective as possible, thereby avoiding any influence on the study’s results. These procedures were designed to guarantee trustworthiness.

Participants’ answers were coded according to their profile (provider or CHW) while maintaining confidentiality. FG responses were coded as Focus Group 1 and Focus Group 2. The citations used to help readers assess the results presented and the credibility of the analysis have therefore been numbered based on the above parameters.

The study and results are reported according to the EQUATOR network’s standards for reporting qualitative research (SRQR) proposed by O’Brien et al. [26] (Appendix 3).

## Results

### General participant characteristics

Table 2 shows the general characteristics of our respondents. Many community members and providers were male, under 50 years of age and married.

**Table 2.** General characteristics of study respondents.

Variable	Number		
	Community		HC providers
	HADC	FG	
**Gender**			
Male	4	12	2
Female	2	4	1
**Age**			
Ender 50 years	6	12	2
≥50		4	1
**Marital status**			
Single	1	1	
Married	5	15	3
**Education**			
Secondary	6	15	
Higher/University		1	3
**Occupation**			
Retailer/Seamstress	1	1	
Farmer/Breeder	1	13	
Civil servant/teacher	4	2	
Healthcare professional			3

(HADC= Health Area Development Committee; FG= Focus Group; HC= Health Center).

### Themes and sub-themes in community ownership of the BPS model

Four main themes were identified in the community ownership of the BPS model of first-level care in Katana HD, DRC: Perceptions of the role of community participation in implementing the BPS Model of care, Community involvement in decision-making and implementation of the BPS Model of care, Challenges and obstacles to community participation in the BPS Model of care, Proposed strategies for ownership and improvement. Table 3 presents these themes, their sub-themes, and their corresponding codes, relating to the three main factors of the conceptual framework.

**Table 3.** Themes, sub-themes, and codes for community ownership of the BPS model.

Themes	Sub-themes	Codes and factors of the conceptual framework
Perceptions of the role of community participation in implementing the BPS Model of Care	Opinion on the organization of community participation and support structures	Community knowledge of the BPS model (OF)
		Role of key partners in community dynamics around the BPS model (OF, MSF)
		Awareness-raising by HADC in support of the BPS model (OF)
	Opinion on holistic care and collaboration	Patient opinion counts and comprehensive care (RF)
Community involvement in decision-making and implementation of the BPS Model of Care	CHW’s role in implementing the model	Periodic meetings with service providers (OF)
		Home visits (OF)
		Referral to psychologist or clinician (OF)
		Follow-up of club activities (OF)
		Preventive and promotional activities (OF, MSF)
	Role and place of patient clubs in implementing the model	Care information sharing (RF)
		Mutual assistance activities in clubs (RF, MSF)
		Collaboration between clubs, HADC and HC in BPS care (RF)
Challenges and obstacles to community participation in the BPS Model of Care	CHW support	Low capacity building (OF)
		Lack of financial motivation (MSF)
	Understanding your role and facing socio-economic obstacles	CHW’s role poorly understood (OF)
		Diffusion of approach and low socio-economic level (OF, MSF)
Strategies for ownership and improvement	Follow-up framework with the various stakeholders and conscientiousness	Involve local leaders in follow-up meetings (OF)
		Reinforce care based on respect for patient needs and empathy (OF)
	Participation in decision-making and psychosocial and financial support	Capacity-building for CHWs in community involvement around the BPS model (OF)
		Involvement of CHWs in relevant exchanges at HC level (OF)
		Importance of the psychologist in BPS care (OF)
		Income-generating activity as a motivating factor (MSF)

(MSF= Motivation/Support factor, OF= Organizational factor, RF= Relational factor) (Figure 2).

## Theme 1: perceptions of the role of community participation in implementing the BPS model of care

### Opinion on the organization of community participation (COMPART) and support structures

The perception of the role of community participation in Katana HD was generally positive. The various HADC members claimed to have already heard of the BPS model of care, and this approach was much more visible in patients with chronic diseases (hypertension and diabetes), who are treated holistically (BPS care). On the other hand, other community members, including CHWs who are not part of the HADC office, confessed to having received no training in this approach.

First of all, as a CHW, I don’t even know this holistic approach to care. I’m not sure what it means to treat someone holistically. This question is complex at our level because we won’t know if we’ve been cared for. (Focus Group 1, 36-year-old)

The elements that best capitalized on this implementation of BPS care were the various meetings organized by providers at the HC level with members of the HADC. Some key partners support interventions for patients with chronic diseases (hypertension and diabetes), thus facilitating the practicability of the BPS model.

Being President of the HADC, I would say that the comprehensive diabetes management approach is gradually extending its intervention, especially in the AHs that are involved in the “Louvain Cooperation” project. (CHW 1, 41-years-old)

In addition, they also believe that the participation of community actors in various activities, including community sensitization, adds value to biopsychosocial care, which helps support the model.

… otherwise, there would even be people who could die even at home because of the lack of sensitization. With that, given that they feel helpless, but given that the HADC or the community gets involved in sensitizing them by showing them that despite insignificant means or lifestyle, the HC will take good care of them… (Provider 3, 48-year-old woman)

### Opinion on holistic and collaboration

Collaboration between the healthcare provider and the care user was noted in the administration and acceptance of care, a partnership environment that puts the patient at the center of everything. Patients had the right to express themselves about the care they received, from this holistic perspective.

He [the patient] can refuse such care, such behavior, and can also give his opinion about his health status… We [the providers] had been told to take the patient on three levels: medical, psychological, and social; what we call the triple look. (Provider 1, 51-years-old)

## Theme 2: community involvement in decision-making and implementation of the BPS model of care

### CHW’s role in implementing the model

The level of community involvement in implementing the BPS model of care was perceived in different ways. On the one hand, that involvement had not yet been optimal, as only a few HAs (4 out of Katana’s 21 HD HAs) had already integrated BPS care, and consequently only a small number of CHWs were involved. On the other hand, where the model was applied, CHWs were sufficiently engaged in its implementation. Regular and periodic meetings at the HC level provided a forum for exchanging the community’s opinions and proposals on the progress of HA.

We ask the community what their impression is of what we’re offering them as a service, and that’s when the CHWs tell us what’s been said in the community, and I give feedback to the team, telling them that this or that behavior isn’t good. That could compromise the whole organization. (Provider 2, 50-years-old)

One way this participation is reconciled with the model was through the practice of home visits, which existed long before, but has been reinforced. These home visits not only facilitated effective follow-up of patients in the community, but also helped disseminate the message of comprehensive care (BPS care) for better acceptance and integration.

As CHWs, I think we have a job to do, because when we make home visits, we have to listen to the community so we can refer them to a clinical psychologist or another provider, depending on how they feel (Focus group 1, 35-years-old woman)

Home visits as follow-up activities were also mentioned in the supervision reports, with a programmatic schedule (twice a week) and a reporting register. These home visits were typically decided during case discussion meetings (Docs 1 and 2). At the Kabushwa HC, these home visits were coordinated by a nurse who had received mental health training and was working as a psychosocial worker. As the HCs in Birava and Mushweshwe do not have a psychosocial worker, a psychologist working at the General Referral Hospital in Katana, was assigned to review specific cases and conduct home visits as needed (Doc 2). In addition to home visits, this role of CHWs also involves patient referrals.

Some community representatives participated in the activities of patient clubs, including diabetic clubs, for follow-up and support in BPS care:

…there are clubs such as the diabetic club, we make sure that the CHW is not missing because it is the one who will follow up on how the activities will take place. And during this meeting in the club, we are allowed to ask how the patient evolves because I do it sometimes… (CHW 4, 33-years-old)

Providers recognized the significant value of community involvement in implementing the BPS model, particularly in the application of preventive and promotional care, which is crucial to this model.

It’s important to involve them because they are the ones who must advise patients every time to respect the rules of hygiene, to practice the exercises, and the conditions required for their health. Together with the relatives who are there, they can exchange experiences to support each other, comfort each other, and it gives psychoeducation… (Provider 1, 51-years-old)

The community recognized the importance that providers place on these preventive and promotional activities during home visits:

…there are times when we make home visits with them (care providers), especially for hypertensive and diabetic patients. They receive many visits from providers, accompanied by CHWs who know the environment. And in visits I’ve been on before, I see the nurse talking to the patients, dietetically, medically and physically, doing sports. (CHW 1, 41-years-old)

### Role and place of patient clubs in implementing the model

Another aspect that helps potentiate the community’s action in caring for patients, particularly those with chronic diseases (such as diabetics and hypertensive) is the presence of patient exchange and sharing clubs Katana’s HD. The organization of patients in solidarity within their clubs contributed to this BPS model by implementing mutual support activities.

And in club meetings [diabetic club], they assist each other, when someone is sick, sometimes they contribute, because they have a social fund … and they go and visit him and give him an amount. And when one of them has another problem, such as grieving in his family, they also help him. (Provider 1, 51-years-old)

These solidarity activities were also supervised at the HC level to ensure their implementation. Club members made home visits to combat social isolation. In addition to the contributions made in these self-managed diabetic clubs, there are also Village Savings and Credit Associations (VSCAs), which the HC supervises. The Kabushwa HC coordinates more than 21 VSCAs, with an average of 30 members per association, including patients with chronic diseases. These VSCAs meet, and their meeting reports are filed at HC. In contrast, the Birava HA also has about 30 VSCAs that were initially supervised by the HC team, as part of a ‘TUSHINDE UJEURI’ project, implemented in 2020–2021. Subsequently, these VSCAs became independent once the project was closed (Docs 1 and 2).

The community recognizes that these diabetic clubs (support groups) are involved in decision-making in BPS care. The diabetic club is even recognized as one of the community participation structures. It is well structured (with a committee) and works closely with HADC and HC.

We all received the same training, the presidents of HADC, the presidents of the diabetic clubs and the local leaders (the prefects, the pastor, the heads of the 10 houses…). In this training we were told that we must work closely together (no secret between HADC and the club, and vice versa). All these things justify our collaboration and we do. (CHW 1, 41-years-old)

## Theme 3: challenges and obstacles to community participation in the BPS model of care

This new approach to care faces significant challenges.

### CHW support

CHWs faced two main challenges in their management:

- Lack of training on the BPS model of care: Community health workers have not received training on this approach. Only HADC presidents have been trained and have just made restitution to other HADC members; this limits their action in the community: *‘We are not (capabilities), that is, we have shortcomings about different activities of the holistic care approach.’ (Focus Group 2, 30-years-old woman)*
- Financial demotivation is forcing CHWs to become less and less involved in the activities of the health facility:

Some CHWs refuse to go to work because they know [that] there is no motivation … We go and get the patients from the community. They come for treatment and they pay. The providers will be paid, but for us who bring these patients there to the HC, there is nothing given, … and that is what discourages us more. But, if there is a motivation, it will help us, and even those who had already given up can come back and continue the work. (Focus Group 1, 41-years-old).

### Understanding your role and facing socio-economic obstacles

For providers, however, CHWs should not expect compensation or motivation for the work they do; instead, they should understand that it is a form of volunteering.

Some people think that being a CHW means that they become salaried workers. When they have just spent 3 months in the community without activities, some give up. They think that going there [to the HC] is getting money while it is volunteering that must take precedence. (Provider 2, 50-year-old)

Moreover, the community believed that this new approach requires the necessary tools to facilitate understanding and dissemination among the population.

…how are we going to work in the community if we don’t have working tools such as image boxes, modules with themes to be developed in the community, so that patients know they have such a disease, but they can still be treated at our HC? … (Focus group 2, 33-years-old).

As a result, there is a need for capacity among all healthcare providers:

…not all providers have been trained in this new way of caring for patients. This approach (care) requires a lot of commitment and energy, and above all the same understanding of the providers… (Provider 3, 48-years-old woman)

The two stakeholders (CHWs and providers) raised the major challenge of the low socio-economic level of the community they face, which seriously hinders access to BPS care.

Most households are not aware of this approach, patients do not have enough money to take care of themselves, like a diabetic who has to enforce his diet but does not have money to buy food… (CHW 3, 35-years-old woman)

## Theme 4: strategies for ownership and improvement

Several proposals and recommendations about holistic care were noted in this BPS model.

### Follow-up framework with various stakeholders and conscientiousness

Overall, a participatory development process involving all stakeholders, including care users (especially patients with chronic diseases), providers, and community workers, is essential for a good understanding, effective collaboration in follow-up, and ownership of the model. The community emphasized the involvement of community leaders in meetings with other stakeholders to follow up on activities supporting the model.

I think we need to invite them [local leaders], to every meeting between us [HADCs], providers, and clubs, so they understand what’s going on. Moreover, they are the ones who must be most involved because they are often with patients. Especially religious leaders, pastors… (CHW 4, 33-years-old)

Providers believe that care in the BPS model should be strengthened by respecting patients’ needs, preferences, and independence, with empathy. A desire for ownership was also raised.

Here at HC, we must take ownership of the approach because it is our mission to care and heal, in support of course with the community that must help us orient patients, and being aware of our profession, we must take ownership of this approach, offering patients comprehensive care by putting ourselves in their shoes, to help the community to have good health. (Provider 1, 51-years-old)

### Participation in decision-making and psychosocial and financial support

Community representatives believe that training on community commitment should be organized with a focus on BPS care, particularly psychosocial support, to ensure good community involvement and collaboration.

The first thing is to train the CHWs, show them how to take care of the population about diseases that can affect them [such as] diabetes, hypertension, other diseases that affect the psychic… have sufficient information on the biopsychosocial approach and there they will be able to sensitize the community well. (CHW 4, 33-years-old)

In addition, the need for full participation in the various decisions relevant at the HC level for HA development was expressed:

We propose that when there are meetings, the caregivers involve us as CHWs to exchange ideas. Let us ask ourselves what is happening in the community, how patients react after being treated, so that we can see how we can help to evolve our HC. (Focus group 1, 31-years-old)

One of the recommendations made in the follow-up reports on PNMNT activities in Katana HD was to strengthen the psychosocial component, specifically by ensuring the regular availability of a psychologist from the General Referral Hospital of Katana, in HAs that lack a psychosocial assistant, to cover the psychological aspects in biopsychosocial care (Doc 1).

For them to be effective and fully involved, community representatives need to be motivated. A better way to address this motivation issue was to establish income-generating activities (IGAs) under the management of community participation structures:

It’s about creating community IGAs that can support themselves. These community IGAs can be managed in the community dynamics, including the CACs according to each village, with a follow-up or supervision from the health district coordination team. (Provider 2, 50-years-old)

## Discussion

From these results, it emerges that community participation in decision-making and ownership of the BPS model of care depend on several factors. Relational factors, such as collaboration between community participation structures (including patient support clubs) and providers, play a crucial role. This collaboration fosters a climate of trust, allowing for better communication and mutual engagement in decision-making through collective planning. Motivational and supportive factors also play a crucial role in the ownership of BPS interventions, facilitating financial autonomy. Organizational factors, including resources and activities, will also contribute to ownership of the BPS model. These factors are outlined in our conceptual framework on community participation in the implementation of the BPS model of care for NCD management (Figure 2), drawing inspiration from Harrison SR & Jordan AM [19].

Four main themes were identified in the results of our study.

Regarding perceptions on the role of community participation in implementing the BPS model of care, it is more visible in Katana HD, particularly among patients with chronic diseases (hypertension and diabetes), who are treated holistically, as also mentioned by Molima et al. [18,27]. Patients have to be involved in their care process. They have the right to refuse unappreciative treatment and report uncomfortable behaviors of healthcare providers during their care process. This freedom of expression creates a climate of trust between the provider and the patient, who is treated as a partner in the care. This partnership enhances patients’ skills and autonomy in making decisions about their care based on their preferences, advice, and interaction with providers [28]. This care partnership should be encouraged in the practice of BPS care.

Regarding community involvement in decision-making and the implementation of the BPS model of care, the reinforcement of home visits ensures close follow-up of patients in compliance with treatment and the implementation of preventive and promotional activities. This fosters a climate of mutual trust between them [29]. VADs also help identify patient needs and reduce the use of healthcare services. A meta-analysis on diabetes management demonstrated the effectiveness of VADs, ensuring glycaemic control and reducing cardiovascular risk factors [30–32]. Other community-based activities, such as referring patients to appropriate care providers [33], contribute to improved control of patients with chronic diseases. All of these activities support the model. The role of diabetic clubs is also recognized as a crucial element in community involvement. These structures provide an environment for the exchange of information and experience between patients, education for the adoption of healthy self-care practices, and the strengthening of interaction between patients and providers [34]. With the establishment of diabetic clubs in various settings, there has been a notable improvement in the quality of life for individuals with diabetes. The BPS model helps patients engage in more dynamic social activities and maintain their self-esteem. Healthcare systems should therefore encourage these social support groups and develop mechanisms to support them in this BPS model.

Speaking of the challenges, the lack of financial motivation, which often rises after participating in a community activity, is one of the main points raised by the respondents. This financial demotivation will have an impact on collaboration and consequently a decrease in the acceptance of the services provided. It should be noted that in the reform of the community health system in the DRC, the consideration of CHWs has shifted from ‘unpaid’ to ‘self-willed.’ The first term is ‘the situation of a person who performs work without obligation, free of charge, without being paid in a community or enterprise.’ The second refers to ‘the situation of a person willingly accepting a mission, a task for the benefit of his or her community. He offers his time without constraint to carry out an activity and eventually receives a reward for his subsistence during this period’ [14]. Referring to this definition, health systems need to consider both monetary and non-monetary incentives to encourage motivation and satisfaction among CHWs. To address, for example, the lack of financial motivation, initiatives such as IGAs or the integration of performance bonuses could be considered to support the engagement of CHWs. Addressing these incentive factors would facilitate their involvement in community activities, ownership and health outcomes, as some studies have shown [35,36].

Capacity building was also identified as a challenge and an improvement strategy. The lack of training for providers and CHWs has an impact on the implementation of the approach, especially since they are unable to assume their responsibilities correctly. Since the BPS model requires multidisciplinary care and community participation, especially in social aspects, regular training on this model is necessary both for the providers (with a focus on humanizing care and multidisciplinary) and for the community (the latter especially for the community component), in the rationalization of chronic diseases in PHC. Capacity building, coupled with quarterly formative supervision by local experts and international partners, will enhance the performance of stakeholders and ensure the quality of services provided [37]. It would also be necessary to orient such supervision in providers’ awareness about humanizing care to ensure that care respects patients’ needs, preferences, and independence. An in-depth analysis of the importance and feasibility of four dimensions identified as fundamental to the integration of BPS care, person-centered (multidisciplinary care, training and education, empowerment of care users, and quality assurance), is crucial to this care process [38].

The involvement of local leaders in implementing the BPS model is also mentioned as a strategy. Although they initially benefited from the training, their involvement was not particularly noticeable. Creating an inclusive follow-up mechanism involving local leaders, including religious leaders, is a necessity. They are even recognized as a key factor in BPS care [39]. This is an important aspect that should be taken into account in the BPS model to provide bio-psycho-socio-spiritual care that enables the improvement of patients’ conditions and well-being [40]. These leaders should then follow up with all the other stakeholders to ensure the success of the interventions. Participatory meetings organized at the HC level, along with collaboration in decision-making, provide this follow-up mechanism and strengthen the partnership between care providers and the community. This partnership approach is well developed elsewhere, with the creation of patient organizations that partner with healthcare systems to achieve the best results in healthcare [41]. These collaborative forums and meetings would provide an opportunity to formalize this partnership and facilitate follow-up on the quality of care.

The need to strengthen patient care by respecting patients’ needs was expressed, with particular attention given to empathy in care, to make it more humanized, from a biopsychosocial perspective. This could then enable us to go beyond biomedical care to address the psychosocial aspects of chronic diseases, as demonstrated by DeHaven MJ et al. [42]. The presence of a psychologist is necessary, to cover the psychological aspects of BPS management. At the primary level, its role is crucial not only in the primary care of psychological pathologies but also in the prevention of various other diseases and education for behavioral change [43].

Chronic diseases are expensive to treat, especially in a country with limited resources, where the population lives at a low socio-economic level, particularly in rural areas, making access to care difficult. In this context, support for activities that guarantee financial autonomy, such as IGAs and/or VSCAs, appears crucial [18]. Solidarity mutual and VSCAs contribute to the development of their members’ socio-economic capital. These types of activities would enable members to support one another when necessary, depending on the available resources, to access various basic services, including health, and to take ownership of the interventions, all under the proper coordination and supervision of the health system. These experiments have yielded positive results elsewhere [44,45].

Using the conceptual framework proposed in this study (Figure 2), we were able to understand the interaction between the different components considered in the BPS perspective of care, aligning with the conceptual model proposed by Harrison SR & Jordan AM [19]. Clear communication between the various stakeholders is essential in this model. Health systems should consider this when managing chronic diseases, particularly in resource-constrained contexts like the DRC. All the above factors should be taken into consideration to promote BPS care in the management of chronic diseases.

The results of our work should reinvigorate the participation of the population and patients in basic community activities, including health, while contributing to universal health coverage and the well-being of the inhabitants. Furthermore, more in-depth research should examine the contribution of community participation to the BPS model of care and its ownership, particularly in person- centered care, through studies incorporating mixed-methodology.

## Study limitations and strengths

Since the qualitative approach used in this study pertains only to one HD, the results may or may not be generalizable outside the local context in which the BPS model is implemented. This study focused solely on community ownership of chronic disease management, without addressing other aspects (analysis of COMPART-related health indicators, etc.). The patient’s role in this model will be explored in a complementary study, which will include observation of practice (home visits, meetings and other promotional activities).

Nevertheless, this study represents one of the few in the sub-Saharan environment, particularly in the DRC, to address community participation in the implementation of the BPS model of care as well as its ownership, at the first-level structures in health systems to improve chronic disease management and quality of care. Although the results were not submitted to an external audit, the detailed description of the context, the structured process for obtaining data (criteria for selecting participants, different data sources…), teamwork to analyze data and interpret results (the coding process, iterative and collaborative), team meetings for debriefing with the supervisor, ensured validity and reliability [46]. Using the conceptual framework proposed in our study, we can understand the interactions of the various components (care providers, the individual, social groups including the family, the entourage, COMPART structures as well as support structures or patient clubs) involved in the provision of care according to this BPS model. The triangulation and the data saturation help to address this research question better.

## Conclusions

This study is one of the few to describe the experiences of community and provider in implementing the BPS model of care, as well as ownership of chronic disease interventions at the first-level facilities in the DRC. Based on the experiences of providers and community representatives, the results showed that this ownership depends on various factors (relational, organizational and motivational/supportive).

The results enabled us to understand the perceptions of both providers and the community regarding the role of community participation in implementing the BPS model of care as well as the interventions that involve the community and enable it to take ownership of the model. However, some challenges and obstacles were identified in this ownership process, and key strategies were proposed to which particular attention should be given, including a participatory stakeholders audit, capacity building on the BPS model for providers and CHWs, and the implementation of income-generating activities to motivate CHWs.

Community ownership of the BPS model is a vital pillar to support effective and resilient chronic disease management, rationalizing it in PHC for better health outcomes. Healthcare systems should consider these identified factors in the policy definition and rationalization process for these diseases by establishing effective coordination mechanisms.

## Supplementary Material

Appendix 1_Healthcare provider interview guide.docx

Appendix 3_SRQR.docx

Appendix 2_Community interview guide.docx

## Data Availability

All data used and analyzed in this study are available from the corresponding author on reasonable request.
