# Supplementary material for: Community ownership of biopsychosocial model of care: a qualitative study in the Katana health district, Democratic Republic of Congo
Source: Glob Health Action. 2025 Sep 4;18(1):2555030. doi: 10.1080/16549716.2025.2555030 (PMC12412321; doi:10.1080/16549716.2025.2555030)
Supplement: Appendix 1_Healthcare provider interview guide.docx [file ZGHA_A_2555030_SM4466.docx]

Healthcare provider interview guide on community ownership of the BPS model

1. What is your perception of the organization of community participation in your area?
2. Do you think that community participation, as currently organized, helps to support biopsychosocial care?
3. Do patients express themselves autonomously in their care, about the therapeutic possibilities offered to them in this holistic care?
4. Do patients discuss their holistic care with support groups (family, friends, patient clubs)?
5. Tell us about the organization and operation of chronic disease support groups in your health area, in connection with BPS management.
6. Do you think that CHWs effectively play their role as community representatives in decision-making at the HC level, with a view to supporting the provision of comprehensive care (BPS)? In what way?
7. Do you involve CHWs in preventive and promotional activities linked to the BPS approach?
8. What challenges have you already identified in this implementation of BPS care (for providers and the community)?
9. What do you suggest to help providers and the community take ownership of the interventions proposed at the CS level, from a biopsychosocial care perspective, in order to offer care that is truly person-centered?
