# Supplementary material for: Community ownership of biopsychosocial model of care: a qualitative study in the Katana health district, Democratic Republic of Congo
Source: Glob Health Action. 2025 Sep 4;18(1):2555030. doi: 10.1080/16549716.2025.2555030 (PMC12412321; doi:10.1080/16549716.2025.2555030)
Supplement: Appendix 3_SRQR.docx [file ZGHA_A_2555030_SM4465.docx]

**Summary Table of Standards for Reporting Qualitative Research (SRQR) as proposed by O'Brien et al.**

| **No.** | **Topic** | **Item** | **Line No in manuscript** |
| --- | --- | --- | --- |
|  | **Title and abstract** | | |
| S1 | Title | Concise description of the nature and topic of the study Identifying the study as qualitative or indicating the approach (e.g., ethnography, grounded theory) or data collection methods (e.g., interview, focus group) is recommended | 1 to 2 |
| S2 | Abstract | Summary of key elements of the study using the abstract format of the intended publication; typically includes background, purpose, methods, results, and conclusions | 29 to 56 |
|  | **Introduction** | | |
| S3 | Problem formulation | Description and significance of the problem/phenomenon studied; review of relevant theory and empirical work; problem statement | 58 to 101 |
| S4 | Purpose or research paradigm | Purpose of the study and specific objectives or questions | 102 to 104 |
|  | **Methods** | | |
| S5 | Qualitative approach and research paradigm | Qualitative approach (e.g., ethnography, grounded theory, case study, phenomenology, narrative research) and guiding theory if appropriate; identifying the research paradigm (e.g., postpositivist, constructivist/ interpretivist) is also recommended; rationale | 176 to 181 |
| S6 | Researcher characteristics and reflexivity | Researchers’ characteristics that may influence the research, including personal attributes, qualifications/experience, relationship with participants, assumptions, and/or presuppositions; potential or actual interaction between researchers’ characteristics and the research questions, approach, methods, results, and/or transferability | 234 to 238 |
| S7 | Context | Setting/site and salient contextual factors; rationale | 107 to 174 |
| S8 | Sampling strategy | How and why research participants, documents, or events were selected; criteria for deciding when no further sampling was necessary (e.g., sampling saturation); rationale | 183 to 195 |
| S9 | Ethical issues pertaining to human subjects | Documentation of approval by an appropriate ethics review board and participant consent, or explanation for lack thereof; other confidentiality and data security issues | 639 to 644 |
| S10 | Data collection methods | Types of data collected; details of data collection procedures including (as appropriate) start and stop dates of data collection and analysis, iterative process, triangulation of sources/methods, and modification of procedures in response to evolving study findings; rationale | 197 to 201, 228 to 232 |
| S11 | Data collection instruments and technologies | Description of instruments (e.g., interview guides, questionnaires) and devices (e.g., audio recorders) used for data collection; if/how the instrument(s) changed over the course of the study | 202 to 227, and 239-241 |
| S12 | Units of study | Number and relevant characteristics of participants, documents, or events included in the study; level of participation (could be reported in results) | 2230 to 232 and 276 to 279 |
| S13 | Data processing | Methods for processing data prior to and during analysis, including transcription, data entry, data management and security, verification of data integrity, data coding, and anonymization/identification of excerpts | 243 to 260 |
| S14 | Data analysis | Process by which inferences, themes, etc., were identified and developed, including the researchers involved in data analysis; usually references a specific paradigm or approach; rationale | 260 to 264 |
| S15 | Techniques to enhance trustworthiness | Techniques to enhance trustworthiness and credibility of data analysis (e.g., member checking, audit trail, triangulation); rationale | 264 to 273 |
|  | **Results/findings** | | |
| S16 | Synthesis and interpretation | Main findings (e.g., interpretations, inferences, and themes); might include development of a theory or model, or integration with prior research or theory | 281 to 289 |
| S17 | Links to empirical data | Evidence (e.g., quotes, field notes, text excerpts, photographs) to substantiate analytic findings | 290 to 475 |
|  | **Discussion** | | |
| S18 | Integration with prior work, implications, transferability, and contribution(s) to the field | Short summary of main findings; explanation of how findings and conclusions connect to, support, elaborate on, or challenge conclusions of earlier scholarship; discussion of scope of application/ generalizability; identification of unique contribution(s) to scholarship in a discipline or field | 477 to 588 |
| S19 | Limitations | Trustworthiness and limitations of findings | 590 to 608 |
|  | **Others** | | |
| S20 | Conflicts of interest | Potential sources of influence or perceived influence on study conduct and conclusions; how these were managed | 637 |
| S21 | Funding | Sources of funding and other support; role of funders in data collection, interpretation, and reporting | 646 |
