# Supplementary material for: Community ownership of biopsychosocial model of care: a qualitative study in the Katana health district, Democratic Republic of Congo
Source: Glob Health Action. 2025 Sep 4;18(1):2555030. doi: 10.1080/16549716.2025.2555030 (PMC12412321; doi:10.1080/16549716.2025.2555030)
Supplement: Appendix 2_Community interview guide.docx [file ZGHA_A_2555030_SM4464.docx]

Community interview guide on community ownership of the BPS model

1. How do you feel community participation is organized in your health area (relationship between the health center (providers) and the community (representatives, leaders, patients)?
2. Have you ever heard of holistic, biopsychosocial care in your health area? What do you think about community participation in this holistic approach to patient care?
3. Do you think that the way patients are cared for at the Health Center takes into account their needs, preferences and independence in this offer of care?
4. Do you think that the health area development committee, the community animation cells and the CHWs play their role effectively as community representatives in decision-making at the health center to promote holistic care? In what way?
5. Do you think that community leaders are also involved in holistic care? If not, how can they be involved?
6. Do you collaborate with support groups (such as patient clubs) in this holistic care? In what way?
7. Do providers involve you in preventive and promotional activities, in line with the BPS approach?
8. What challenges/difficulties do you encounter in supporting the biopsychosocial approach to care?
9. What do you propose to do to take ownership of the various interventions proposed at the Health Center level to improve the provision of quality, person-centered care that takes into account BPS aspects?
